# Large-Scale Assessment of Bioinformatics Tools for Lysine Succinylation Sites

**DOI:** 10.3390/cells8020095

**Published:** 2019-01-28

**Authors:** Md. Mehedi Hasan, Mst. Shamima Khatun, Hiroyuki Kurata

**Affiliations:** 1 Department of Bioscience and Bioinformatics, Kyushu Institute of Technology, 680–4 Kawazu, Iizuka, Fukuoka 820-8502, Japan; shammistat85@gmail.com (M.S.K.); kurata@bio.kyutech.ac.jp (H.K.); 2 Biomedical Informatics R&D Center, Kyushu Institute of Technology, 680-4 Kawazu, Iizuka, Fukuoka 820-8502, Japan

**Keywords:** lysine succinylation, sequence analysis, machine learning, tool development, feature descriptor

## Abstract

Lysine succinylation is a form of posttranslational modification of the proteins that play an essential functional role in every aspect of cell metabolism in both prokaryotes and eukaryotes. Aside from experimental identification of succinylation sites, there has been an intense effort geared towards the development of sequence-based prediction through machine learning, due to its promising and essential properties of being highly accurate, robust and cost-effective. In spite of these advantages, there are several problems that are in need of attention in the design and development of succinylation site predictors. Notwithstanding of many studies on the employment of machine learning approaches, few articles have examined this bioinformatics field in a systematic manner. Thus, we review the advancements regarding the current state-of-the-art prediction models, datasets, and online resources and illustrate the challenges and limitations to present a useful guideline for developing powerful succinylation site prediction tools.

## 1. Introduction

Lysine succinylation is an evolutionarily conserved posttranslational modification (PTM) known to be involved in the regulation of diverse cellular process [1,2,3,4,5,6,7]. The succinylation process modifies a target protein with a succinyl group (–CO–CH_2_–CH_2_–CO_2_H), which is transmitted from succinyl-CoA to the specific α-amino group of a lysine residue [8,9,10,11,12]. The succinylation firstly was discovered in histone protein [13], and its regulatory role has been examined through the gene expression regarding chromatin reorganization [14,15,16]. Nevertheless, the published studies have provided little information regarding the enzyme which catalyzes histone lysine succinylation [17,18,19]. In fact, it is unclear whether this reaction is enzymatic or not [8,9,20]. In addition to histones, the succinylated proteins were found in the cytoplasm, nucleus, and mitochondria [7,21,22,23,24], indicating that lysine succinylation controls a variety of biological functions [14,18,25,26]. Lysine succinylation in HeLa cells induced different diseases via histone proteins, including UV-induced stress and cancer [12,27,28,29,30,31,32,33,34]. Therefore, identification of succinylation sites is a key to understanding the functional proteins.

A few years ago lysine succinylation was identified as a protein modification [2,3,25]. This modification can make notable alterations in protein function and structure regulation [3,13,35,36,37]. It can also participate in regulating many biological processes such as calorie restriction and metabolisms [38,39,40,41,42,43,44]. The identification of protein succinylation sites is a crucial topic in cellular pathology and physiology, which may provide valuable information for biomedical research and drug development. In recent years, high-throughput methods with mass spectrometry and succinylation enrichment analysis have been extensively implemented to identify lysine succinylation in several organisms [1,2,7,22,25,37,45,46,47,48,49]. A large-scale protein lysine-succinylated sites have been verified by experimentally in both prokaryotes [7,24,50,51] and eukaryotes [2,24,25,47]. Despite great advances through experimental investigation, the conventional experimental approaches are still difficult and time-consuming tasks [5,7,44,52,53]. Computational methods for succinylation site prediction are highly needed before experimental validation.

**Figure 1:**
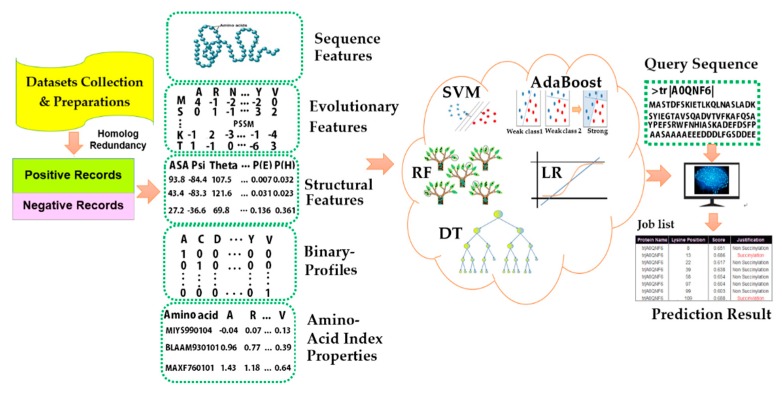
An overview of current computational prediction algorithms of succinylation sites.

Our objective is to provide the useful and practical guidelines for the prediction of protein succinylation and to illustrate which predictor performs the best, whether the existing prediction model can be improved, and which features significantly contribute to prediction accuracy. We have assessed the performance of two different statistical methods: support vector machine (SVM) and random forest (RF) with five major types of descriptors. We also assess the performances of the individual and combined features with statistical significance tests, illustrating their contribution to the prediction accuracy. A synopsis of the existing computational approaches for lysine succinylation prediction is presented in Figure 1.

## 2. Existing Prediction Models

Nowadays, several machine learning-based predictors have been employed to identify succinylation sites [54,55,56,57,58,59,60,61,62,63,64,65,66,67,68,69,70]. The SucPred [54] is the first succinylation site predictor, which was established by Zhao et al. in 2015 through different encoding descriptors, including position amino acids weight composition, van der Waals volume normalized, grouped weight-based encoding, and auto-correlation functions, via SVM. By using SVM, Xu et al. developed iSuc-PseAAC [55] that implemented a composition of pseudo-amino acids (PseAAC) scheme. The SuccFind [56] predictor was established by Xu et al. which considered several amino acid-based composition encodings, including amino acid composition (AAC), k-space amino acid pairs (CKSAAP), and amino acid index (AAindex) through a feature selection algorithm. Two prediction tools of iSuc-PseOpt [70] and pSuc-Lys [61] were constructed by Jea et al., based on the PseAAC descriptor via a RF classifier. The SucStruct [58] and Success [67] predictors were developed by Lopez et al. based on the secondary structure-based features (SF) with decision trees (DT) algorithm. Dehzang et al. constructed two prediction tools of PSSM-Suc [57] and SSEvol-Suc [66] with a DT classifier by using evolutionary- and sequence-based features [67,68]. Hasan et al. developed the SuccinSite [59], SuccinSite2.0 [62], and GPSuc [65] predictors with the RF classifiers by integrating multiple sequence features. The SuccinSite2.0 [62] and GPSuc [65] predictors implemented different species-specific classifiers and integrated them. Until now, the GPSuc is one of the most updated predictors. On the other hand, abovementioned existing methods differ in various aspects, such as training and test datasets used, sliding window sizes and algorithms preferred, a ratio of positive versus negative samples, categories of sequence features encoded, and generality of whether the predictive classifiers are universal or species-specific. In addition, there have been distinct differences in terms of practical aspects of the web server implementation, adjustability of prediction inflexibility thresholds, support of batch predictions and computational efficiency. With various succinylation site predictors becoming available, comprehensive comparison of the strengths and weaknesses of them are essential. This comparison may reveal difficulties and guide improvement toward efficient succinylation site predictors.

A lot of focus has been placed on research of protein succinylation with an increase in databases [59,71,72]. The SuccinSite database records 4411 experimentally identified succinylation proteins with 12,456 lysine succinylation sites for different species [59]. It should, however, be noted that the succinylation proteins overlap with other modifications due to some exhibiting dual properties. Recently many studies have suggested that lysine succinylation extensively overlaps with acetylation [25,27,42,63,68,73,74,75,76].

To date, 12 methods were analyzed, i.e., SucPred [54], iSuc-PseAAC [55], SuccFind [5,6], iSuc-PseOpt [70], pSuc-Lys [61], SucStruct [58], PSSM-Suc [57], SuccinSite [59], SSEvol-Suc [66], SuccinSite2.0 [62], Success [67], and GPSuc [65] (Table 1., The SucPred used highly unbalanced (i.e., 1436 positive and 18,958 negative samples) training datasets, derived from the CPLM (http://cplm.biocuckoo.org) database [71]. For testing models, they used 250 positive samples but did not consider any negative samples. The pSuc-Lys, iSuc-PseAAC, and iSuc-PseOpt used 1167 positive and 3553 negative samples as the training dataset from the CPLM database but did not consider any independent datasets. The SucFind used 2713 positive and 23,598 negative samples as the training dataset from the CPLM database but did not consider any independent sets. The PSSM-Suc used 1782 positive and 1872 negative samples as the training dataset but did not consider any independent samples. The Success [67], SucStruct [58] and SSEvol-Suc [66] used a balanced training dataset (1782 positive and 1872 negative samples) from the CPLM database but did not consider any independent samples. In addition, few existing predictors have updated the latest datasets [59,65].

## 3. Datasets Collection and Preparation

### Positive and Negative Samples

**Figure 2:**
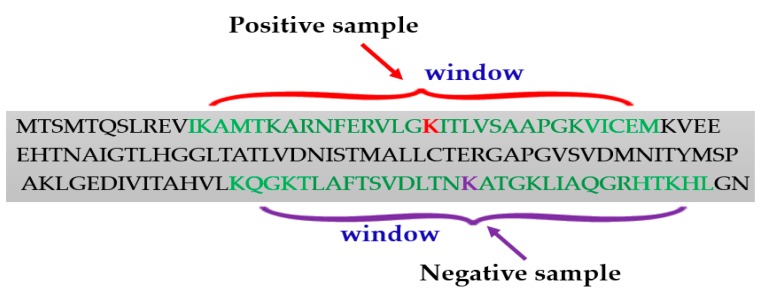
Window selection procedure for generating positive and negative samples.

Generating the positive and negative samples from the protein sequences is an important step for lysine succinylation sites prediction. Usually, the positive samples were collected based on the experimentally verified lysine (K) residues. The sequence window strategy was applied to construct the positive samples. The fragment windows were the sequences of the peptide with a lysine residue to be succinylated in the center. To accurately predict succinylation sites, analysis of flanking residues in the window fragment is important, because a very small number of residues would miss valuable evidence and a large number of them may introduce unavoidable redundancy. For example, to select the window fragments of 31 (±15), the length of the full sequence of proteins was inputted; for the fragment window model, a window size of 31 was fixed so that the lysine residue is centered (Figure 2). Most of the researchers have tested different window fragments to enhance predictive performance in succinylation site prediction (Table 1).

To generate a set of fragment windows that are regarded as negative samples are very challenging. There is no standard method to generate the negative samples. Researchers typically considered the experimentally identified succinylated lysines as positive samples, while they regarded all the remaining lysine residues as negative instances. Nonetheless, some negative samples may be positive are generated by experimental errors, which decreases prediction accuracy.

Recently thousands of succinylated proteins and their sites have been identified experimentally from diverse species including *Homo sapien**s*** (*H. sapiens*), *Saccharomyces cerevisiae* (*S. cerevisiae*), *Mus musculus* (*M. musculus*), *Toxoplasma gondii* (*T. gondii*), *Histoplasma capsulatum* (*H. capsulatum*), *Mycobacterium tuberculosis* (*M. tuberculosis*), *Escherichia coli* (*E. coli*), *Solanum* *lycopersicum* (*S. lycopersicum*), and *Triticum aestivum* (*T. aestivum*) [7,22,37,47,59]. To examine the species-specific datasets, we collected the datasets of nine species and removed redundant sequences with a 30% similarity cutoff using CD-HIT [77] and recorded them at http://kurata14.bio.kyutech.ac.jp/GPSuc [65]. A statistic of the training and independent datasets is shown in Table 2.

## 4. Algorithms of Predicting Lysine Succinylation Site

Many machine learning algorithms such as RF, SVM, adaptive boosting (AdaBoost), and DT have been employed to predict succinylation sites, while the two machine learning algorithms of SVM and RF are intensively used (Table 1). Employed machine learning algorithms are briefly explained as follows.

### 4.1. Random Forest

In protein bioinformatics research, RF is a well-established and extensively used machine learning algorithm [62,65,78,79]. RF works as a collective and supervised decision classifier, which ‘votes’ for one of the two classes, either positive or negative samples. The RF algorithm is very straightforward and does not produce any bias results. However, it is necessary to select the optimum number of decision trees. In this review, to examine the selected, individual descriptors, we used 1000 decision trees via 5-fold cross-validation (CV) test to validate the method performances by using a package of R software (https://cran.r-project.org/web/packages/randomForest/).

### 4.2. Support Vector Machine

SVM is another machine learning algorithm and broadly used in protein bioinformatics research [54,55,56,57,80]. Various kernel function including the linear/polynomial/sigmoid and Gaussian radial basis function were used to develop SVM models. A critical point is the optimization of parameters. Prior to model construction, it is recommended to optimize SVM parameters, which affect the prediction performance dramatically. In this review, we used the SVM^light^ (http://svmlight.joachims.org) package to examine the individual features with default parameters.

### 4.3. Adaptive Boosting

AdaBoost works as a meta-classifier that is frequently used to classify binary samples [66]. This algorithm iteratively adjusts weight values to decrease the misclassified samples until the weight values do not change.

### 4.4. Decision Trees

DT is a non-parametric machine learning approach and generates logical diagrams by learning specific rules [57,58]. On the other hand, DT sometimes causes biased prediction for high dimensional datasets.

## 5. Motif Conservation of Species-Specific and Generic Succinylation Sites

**Figure 3:**
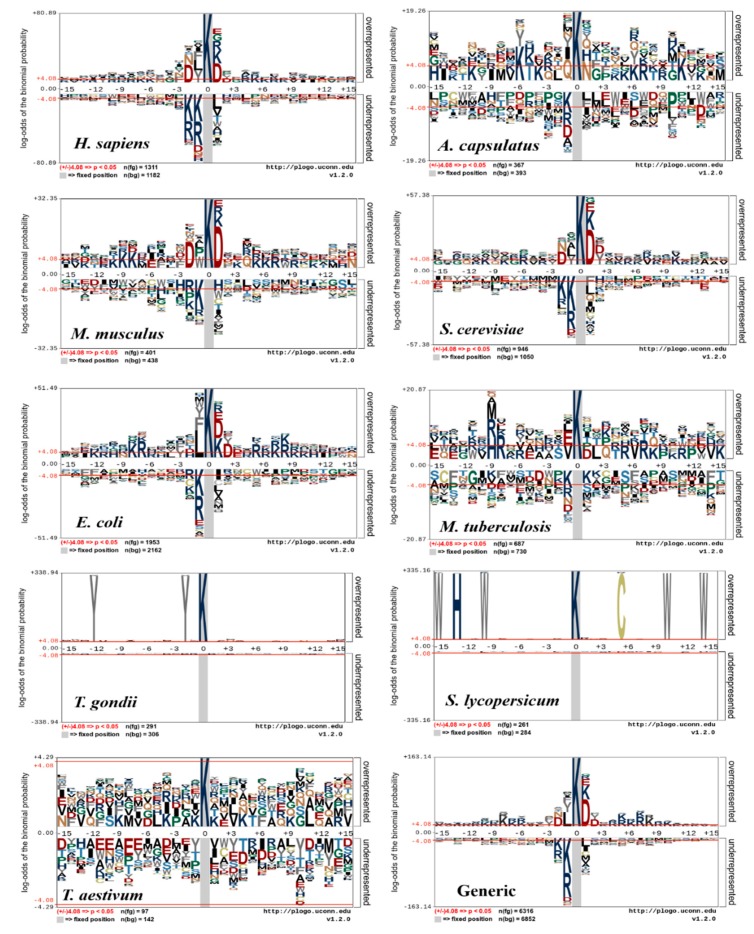
pLogo graphs of the sequences with the centered succinylation sites. Nine species-specific datasets of *H. sapiens*, *H. capsulatum*, *M. musculus*, *E. coli*, *M. tuberculosis*, *S. cerevisiae*, *T. gondii*, *S. lycopersicum* and *T. aestivum* (https://plogo.uconn.edu/) and their combined (generic) datasets are used. The significantly enriched/depleted amino acid residues (student *t*-test, *p* < 0.05) are shown.

The sequence motif conservation surrounding the succinylation sites could partly be illustrated for the different species datasets. To reveal succinylation site sequences of 9 different species, a pLogo (https://plogo.uconn.edu/) software was used as shown in Figure 3 [81], which classifies and displays significant differences of succinylated vs non-succinylated sites by position-specific amino acid compositions on the sequence fragments (±15). At each position of pLogo graphs, over- or under- X-axis amino acids were plotted, where X denotes each amino acid residue [59,65,78]. The height of the corresponding residue letter of positive (if over-represented) or negative samples (if under-represented) were harbored. The cumulative percentages of these over-/under-represented residues were reported in the label of Y-axis. Consequently, the amino acids above the X-axis indicated frequently detected residues around succinylation sites. In Figure 3, the upper portion displays a set of positive samples and the middle portion displays consistent residues, while the lower portion shows depleted amino acids.

Since the sequence motifs for *H. sapiens*, *S. cerevisiae*, and *M. musculus* resembled each other (Figure 3), an *H. sapiens* succinylation site tool could identify succinylation sites for *M. musculus*, and *S. cerevisiae* and the reverse is also true. The sequence patterns of succinylated proteins around *H. sapiens*, *M. musculus*, *H. capsulatum*, *S. cerevisiae*, and *E. coli* are widely distributed than the other four species. It was observed that charged amino acids (K, R, and D) were significantly enriched at positions (−10, −9, −8, −7, −6, −5 −2; +2, +4, +5, +6, +7, and +10) for *H. sapiens*, *M. musculus*, *H. capsulatum*, *S. cerevisiae*, and *E. coli* models. In *S. lycopersicum*, *M. tuberculosis*, and *T. aestivum* species, the neutral amino acids (C, F, G, and S) were significantly depleted. In *S. cerevisiae* and *T. gondi*, some of the charged residues (D, K, and R) were over- and under-represented. In addition, neutral amino acids (S, Q, and C) were frequently distributed around the succinylation sites and most of the specific amino acid positions were not significantly enriched/depleted except for *S. lycopersicum*, *T. gondii*, and *T. aestivum*. While the generic model seems to have some sequence motifs, it is clearly shown that the sequence motifs are species-specific. Therefore, the generic model may result in incomplete or erroneous information to a query sequence. Hasan et al. suggested that the surrounding succinylation sites vary, depending on species [65] and the species-specific classifiers are necessary to identify the succinylation sites, as well as developers of other PTM site predictors for ubiquitination [82], acetylation [83,84], methylation [85], phosphorylation [86,87], and malonylation [88].

## 6. Important Descriptors for Predicting Succinylation Sites

Feature extraction is one of the most important and challenging steps, enabling the accurate prediction of lysine succinylation sites. Ideally, the features can clearly distinguish succinylated sites from random lysine sites. In previous studies, different types of features were adopted to distinguish the succinylated sites from non-succinylated sites. The frequently used features are AAindex, ACF, EBGW, VDWV, WAAC, AAC, CKSAAP, PseAAC, Binary, SF, PSSM, pCKSAAP and some structural features (SFs) (Table 3). These major feature types include (1) protein sequence features, (2) evolutionary features, (3) protein physicochemical properties, (4) structural features, and (5) binary profile annotations.

To develop a statistical predictor, an effective mathematical expression is needed to formulate the protein or peptide samples [89,90,91,92]. Composition analysis of proteome-wide amino acids can describe the particular information of a specified organism, since the organism manages to reduce the protein synthesis cost by adjusting their residue contents under specific growth conditions [19,93]. Therefore, sequence information was valuable to develop species-specific succinylation predictors. To transform protein or fragment sequences into numeric vectors, orthogonal binary coding [59,62], AAindex [65], PseAAC [55,61,70] were measured. To accesses the positional information of amino acids around the positive and negative samples, the WAAC [54], ACF [54], and VDW [54] were introduced. Moreover, to introduce the amino acids frequency information in fragment sequences, the pCKSAAP [62,65] and CKSAAP [56,59] schemes were used. To fix the length of the sequence, AAindex encoding is particularly suitable [59,62,65]. To identify the conserved residues at the specific sequence, evolutionary information is an important characteristic [57,65], because the conserved residues are always functionally relevant [62]. Since the SF is far more conserved than the sequence, SF encoding could be a valuable indicator to identify the function of succinylation proteins [58]. To make an effective prediction model, optimization of incorporative feature methods is typically crucial. The SuccinSite used a linear combination of different features with weight values [59]. Recently, the outputs of distinct features have been combined using a logistic regression (LR) algorithm [65,94]. These two models can be integrated for further enhancement of accuracy of succinylation site prediction.

## 7. Features Assessment of Species-specific Succinylation Sites

To classify the succinylation and non-succinylation samples, machine learning algorithms have been effectively employed (Table 1). A majority of succinylation site predictors used conditional RFs [57,58,59,61,62,70], while a few of them used SVM classifiers [54,55,56]. Therefore, we chose these two machine learning algorithms due to their successful implementation. We also measured the area under the ROC curve (AUC). Table 4 summarizes the optimal performances with respect to 31 window sequences by the RF and SVM classification algorithms.

Twelve types of feature descriptors were employed in the previous succinylation predictors (Table 3). We investigated whether they are effective in prediction of the nine species-specific models and selected five major descriptors of CKSAAP, AAindex, Binary, PseAAC, and pCKSAAP (the other seven descriptors were not effectively used). A five-fold CV test on the training dataset and a test on the independent dataset were performed to assess the prediction performance by the five selected feature descriptors (Table 4), where the employed datasets are shown in Table 2. The top two features for *H. sapiens*, *M. musculus*, *H. capsulatum*, and *E. coli* were pCKSAAP and CKSAAP for training dataset. On the other hand, in the independent dataset, the AAindex and binary performed better. For the *M. tuberculosis* dataset, the top two features were pCKSAAP and CKSAAP in both of training and independent datasets. In the *S. cerevisiae* dataset, the top descriptor was pCKSAAP. In the *T. gondii* and *T. aestivum* datasets, CKSAAP, pKSAAP, and PseAAC encoding schemes were important. It is intriguing that, in the *S. lycopersicum* dataset, positional encodings of Binary, AAindex, and PseAAC were essential for the independent test. The pCKSAAP was an effective encoding feature that describes long- and short-range interfaces of amino acids within a protein or a sequence window [95,96,97,98], achieving best prediction results on *M. tuberculosis, H. sapiens, M. musculus, H. capsulatum, S. cerevisiae, E. coli,* and *T. aestivum* species for training datasets. The performance comparison indicated that the RF algorithm was the best for almost all the species datasets, followed by the SVM.

## 8. Comparative Analysis of Different Predictors

**Figure 4:**
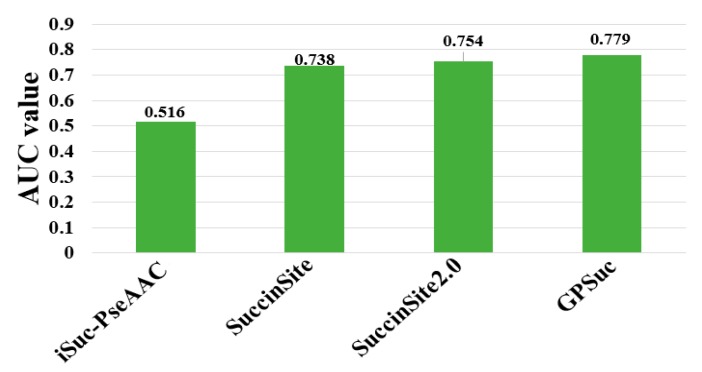
Performance comparison of generic succinylation site prediction models on an independent dataset.

The performances of existing tools were compared by using different criteria as shown in Table 1. Note that it is difficult to exhaustively compare the analytical results obtained from different algorithms, because they use diverse assessment procedures for training and independent datasets and ratios of positive and negative samples. Although many predictors are not publicly accessible, including Success, SSEvol-Suc, SucPred, SucPred, pSuc-Lys, iSuc-PseOpt, SuccFind, SucStruct [58], and PSSM-Suc [57], only four of succinylation predictors of iSuc-PseAAC, SuccinSite, SuccinSite2.0, and GPSuc are publicly available and user-friendly. An independent dataset was constructed to make a fair comparison based on our previously published articles [65]. The dataset consisted of 254 positive and 2977 negative samples (http://kurata14.bio.kyutech.ac.jp/GPSuc) [65]. Figure 4 shows that the prediction performance of the four predictors with respect to 124 proteins. The top-performing SuccinSite2.0 and GPSuc with the AUC value of 0.754 and 0.779, respectively.

Recently the GPSuc and SuccinSite2.0 predictors have made an effort to establish the species-specific classifiers [62], while the others combined the data of each species into a generic model. Many predictors other than SuccinSite [59], SuccinSite2.0 [62], and GPSuc [65] were not validated by using independent data (Table 1).

## 9. The Online Employment Services

For biologists, web application or a standalone software package is required. There were 12 web services developed along with research publication; however, most of them are not available for public. The exiting tools were compared under the following conditions: (i) whether the existing web employment supports batch prediction; (ii) whether the scheme has the binary or probability scores; In Table 1, comprehensive information was summarized for all the existing tools. Among all the implementations, Success, PSSM-Suc and SucStruct did not provide web-services to implement their prediction models. The pSuc-Lys, SSEvol-Suc, and Suc-PseOpt predictors did not fulfill some criteria regarding sequence fragment position, prediction scores, and thresholds information. On the other hand, users cannot submit more than 100 sequences to the pSuc-Lys and Suc-PseOpt servers. The iSuc-PseAAC and Success servers did not attach the all prediction succinylation scores in the final output page. Users can get more satisfactory results from the SuccinSite, SuccinSite2.0, and GPSuc in a FASTA format. In the GPSuc user can select classifiers for nine species and their combined species. The GPSuc includes nine examined species classifiers and illustrated better performances than the SuccinStie2.0. The prediction output of the GPSuc, SuccinSite, and SuccinSite2.0 contains four items: protein name, predicted lysine position, expectation score, and explanation of succinylation sites. In the viewpoint of users, the prediction model should contain at least the position of the anticipated succinylation sites, sequence fragments, and probability scores, or assessment of the predicted result. In addition, it is obligatory that the predictor should provide flexibility modification to the output page of the provided stand-alone software or online servers. Particularly user control of the prediction stringency is essential for spreading predictors because users are interested in the prediction scores with an assured threshold.

## 10. Perceptions for Prediction Models

Sequence redundancy is an essential problem to consider prior to model assembly since the performance of the predictive models might be overestimated by overfitting of the training dataset and lead to poor scalability and performances on independent datasets. In succinylation prediction, most of the developers conducted the redundancy of sequence prior to model assembly. The CD-HIT (http://weizhongli-lab.org/cd-hit) [77,99] and BLAST algorithm (blastclust) (http://nebc.nox.ac.uk/bioinformatics/docs/blastclust) [100] are extensively used to eliminate data redundancy. The CD-HIT software is very popular for deleting the homolog sequences; however, this framework is a heuristic, i.e., it can have biases on the redundancy level model [101]. Recently, Martin and Johannes introduced the Linclust software (https://github.com/soedinglab/mmseqs2) [102] to reduce the compositional bias correction on the sequences, while advanced algorithms are still necessary. To reflect the ratio of succinylation and non-succinylation samples in the training data set is another problem. Usually, non- succinylation sites expressively outnumber the succinylation sites. Hence, a succinylation training dataset should be generated by using reliable and nonbiased methods. To choose the ratio of non-succinylation ratio samples to positive samples, a random selection procedure is often piloted.

Some prediction tools use small datasets to train their simulations, resulting in poor estimate performance when verified with the independent dataset [59,62]. For instance, an early study of the iSuc-PseAAC did not achieve good performance on the independent test dataset due to the limited training dataset (Figure 4). Through the developments in high-throughput sequencing with mass spectrometry analysis, a large number of succinylation sites are being identified and their associated databases are frequently updated. Many succinylation sites that were overlooked by previous studies are now experimentally verified as positive samples, i.e., the old versions of the database include a number of false negative samples. This indicates that the prediction models developed based on the old version database can be improved by using up-to-date succinylation samples. To extrapolate future unknown data, we should increase the number of non-redundant succinylation samples and use them as an independent dataset to validate the prediction models.

The motifs of succinylation proteins may significantly differ in diverse species, as shown in Figure 3. Nevertheless, all the existing predictors other than SuccinSite2.0 and GPSuc ignored the differences among species and combined all species models into a generic one. From now on, a computational method should consider species-specific classifiers. The current prediction tools are established individually based on sequence or secondary structural information. In future analysis, with an increase in tertiary structural information of succinylation samples, it is effective to employ such a structural descriptor [103]. Finally, it is required to present software applications or web servers so that users can easily access prediction models.

To reveal the significant information on the PTMs, graphical logos are widely used that give position-specific information (i.e., conserved patterns or motifs information) of amino acids. Several software packages are implemented to visualize the sequence motifs, such as pLogo [81], WebLogo [104], and iceLogo [105]. The existing algorithms highlighted the characters of amino acids that are enriched (i.e., occur more frequently than expected) and depleted (i.e., occur less than expected). However, the resulting plots sometimes suffered visual disorder, which makes principal sequence patterns ambiguous. Therefore, the next generation sequence logo needs to generate more suitable models for the efficient visualization of sequence motifs.

## 11. Conclusions

To assess the currently available succinylation site prediction tools, we comprehensively compared the predictor performances using an independent dataset. The predictive capabilities of combinations of different descriptors were evaluated to explore the optimal combination. In living cells, combining experimental and computational approaches will accelerate the buildup of our understanding on protein succinylation and hence support exploration of the consistent controlling networks. This review has designated that a large volume of lysine-succinylation site analyses is being carried out and explained the details in the employed datasets, motif conservation, encoding schemes, and machine learning algorithms. Moreover, we described limitations of current methodologies for prediction of lysine succinylation and provided perceptions into dataset assembly processes, model updates, and performance improvements.

## Figures and Tables

**Table 1:** Summary of the reviewed predictors for lysine succinylation sites.

Tools	SucPred	iSuc-PseAAC	SuccFind	iSuc-PseOpt	pSuc-Lys	SucStruct	PSSM-Suc	SuccinSite	SuccinSite2.0	SSEvol-Suc	Success	GPSuc
Species	Generic	Generic	Generic	Generic	Generic	Generic	Generic	Generic	Generic and Species-specific	Generic	Generic	Generic and Species-specific
Web-server link	http://59.73.198.144:8088/SucPred/	http://app.aporc.org/iSuc-PseAAC/	http://bioinfo.ncu.edu.cn/SuccFind.aspx	http://www.jci-bioinfo.cn/iSuc-PseOpt	http://www.jci-bioinfo.cn/pSuc-Lys	https://github.com/YosvanyLopez/	https://github.com/YosvanyLopez/PSSM-Suc	http://systbio.cau.edu.cn/SuccinSite/	https://biocomputer.bio.cuhk.edu.hk/SuccinSite2.0/	https://github.com/YosvanyLopez/SSEvol-Suc	https://github.com/YosvanyLopez/Success	http://kurata14.bio.kyutech.ac.jp/GPSuc/
Working server	No	Yes	No	No	No	No	No	Yes	Yes	No	No	Yes
Machine learning	SVM	SVM	SVM	RF	RF	DT	DT	RF	RF	AdaBoost	SVM	RF and LR
Dataset size (Protein/succinylated)	897/2511	896/2521	1044/2938	896/2521	896/2521	670/1782	670 / 1782	2322/5004	2322/5004	670/1782	670/1782	2322/5004
Training (Pos/Neg)	1436/18,958	1167/3553	2713/23598	1167/3553	1167/3553	1782/1872	1782/1643	4750/9500	4750/9500	1782/1872	1782/1872	4750/9500
Independent (Pos/Neg)	250/-	-	-	-	-	-	-	254/2977	254/2977	-	-	254/2977
Homolog redundancy	35%	40%	30%	40%	40%	40%	40%	30%	30%	40%	40%	30%
Window size	from −9 to +9	from −7 to +7	from −10 to +10	from −15 to +15	from −15 to +15	from −15 to +15	from −15 to +15	from −13 to +13	from −20 to +20	from −15 to +15	from −15 to +15	from −20 to +20
Adjusted batch prediction	NO	No	No	No	No	No	No	Yes	Yes	No	No	Yes
Processing time for a protein	-	Within 20 s	-	-	-	-	-	Within 20 s	Within 5 min	-	-	Within 5 min

**Table 2:** Statistics of the positive and negative samples of nine species-specific datasets used in this study.

Species	Datasets	Positive Samples	Negative Samples
*H. sapiens*	Training	1351	2702
	Independent	54	2004
*M. musculus*	Training	414	828
	Independent	24	679
*E. coli*	Training	1942	3884
	Independent	289	1381
*M. tuberculosis*	Training	699	1398
	Independent	61	242
*S. cerevisiae*	Training	961	1922
	Independent	90	1423
*T. gondii*	Training	282	564
	Independent	26	261
*S. lycopersicum*	Training	242	484
	Independent	33	274
*A. capsulatus*	Training	332	664
	Independent	50	591
*T. aestivum*	Training	113	226
	Independent	32	309

**Table 3:** Statistics of feature encoding schemes used in the aforementioned succinylation site prediction tools.

Encoding Types	Genetic Explanation	References
AAindex	Based on the AAindex indices database, the encoding scheme of AAindex reveals the biochemical properties of the sequences.	[56,59,62]
ACF	The auto correlation function features for surrounding succinylation sequences.	[54]
EBGW	Coding based on grouped weight of physicochemical properties of sequences surrounding succinylation sites.	[54]
VDWV	Van der Waals volume properties of surrounding succinylation sequences.	[54]
WAAC	Position weight amino acid composition of surrounding succinylation sequences.	[54]
AAC	The amino acid composition characterizes the specific state of the surrounding succinylation sequences.	[65]
CKSAAP	The CKSAAP encoding represents the short sequence motif information in surrounding succinylation sites.	[56,59]
PseAAC	The pseudo amino acid composition reflects a vectorized sequence-coupling model of surrounding succinylation sites.	[56,61,70]
SF	The predicted structural feature reflects the structural properties of protein in surrounding succinylation sites.	[66]
Binary	The position-specific information measured by binary profile for the curated sequences.	[59,62,65]
PSSM	The PSSM exposes the evolutionary information from the sequences.	[57]
pCKSAAP	The pCKSAAP reflects the sequence patterns and evolutionary information from the query sequences.	[62,65]

Data of Table 1 is used.

**Table 4:** Performance of five major types of features for the training and independent datasets.

Methods		Training		Independent	
*H. sapiens*		RF	SVM	RF	SVM
	pCKSAAP	0.856	0.838	0.695	0.691
	CKSAAP	0.816	0.831	0.677	0.663
	AAindex	0.739	0.728	0.759	0.755
	Binary	0.767	0.754	0.822	0.809
	PseAAC	0.819	0.822	0.658	0.649
*H. capsulatum*	pCKSAAP	0.789	0.792	0.638	0.634
	CKSAAP	0.788	0.783	0.619	0.607
	AAindex	0.712	0.722	0.658	0.666
	Binary	0.713	0.698	0.665	0.647
	PseAAC	0.759	0.743	0.612	0.614
*M. musculus*	pCKSAAP	0.801	0.788	0.637	0.634
	CKSAAP	0.777	0.767	0.646	0.651
	AAindex	0.648	0.655	0.679	0.672
	Binary	0.639	0.641	0.677	0.659
	PseAAC	0.711	0.722	0.609	0.611
*E. coli*	pCKSAAP	0.769	0.761	0.679	0.684
	CKSAAP	0.773	0.782	0.646	0.631
	AAindex	0.719	0.721	0.633	0.619
	Binary	0.689	0.674	0.619	0.607
	PseAAC	0.733	0.734	0.608	0.603
*M. tuberculosis*	pCKSAAP	0.708	0.712	0.688	0.679
	CKSAAP	0.689	0.675	0.664	0.671
	AAindex	0.667	0.658	0.656	0.655
	Binary	0.629	0.617	0.639	0.634
	PseAAC	0.643	0.634	0.629	0.617
*S. cerevisiae*	pCKSAAP	0.882	0.869	0.776	0.772
	CKSAAP	0.879	0.863	0.752	0.744
	AAindex	0.742	0.733	0.759	0.749
	Binary	0.741	0.745	0.798	0.787
	PseAAC	0.790	0.768	0.699	0.675
*T. gondii*	pCKSAAP	0.834	0.836	0.657	0.666
	CKSAAP	0.826	0.822	0.655	0.638
	AAindex	0.726	718	0.663	0.647
	Binary	0.744	0.745	0.679	0.671
	PseAAC	0.801	0.788	0.678	0.664
*S. lycopersicum*	pCKSAAP	0.842	0.836	0.649	0.642
	CKSAAP	0.833	0.824	0.648	0.637
	AAindex	0.753	0.765	0.644	0.629
	Binary	0.729	0.722	0.637	0.631
	PseAAC	0.801	0.783	0.678	0.658
*T. aestivum*	pCKSAAP	0.822	0.826	0.649	0.654
	CKSAAP	0.821	0.811	0.638	0.634
	AAindex	0.736	0.734	0.604	0.611
	Binary	0.726	0.719	0.612	0.596
	PseAAC	0.778	0.769	0.632	0.628

AUC values are used to assess the prediction performance.
